# Efficacy and safety of endoscopic sleeve gastroplasty and laparoscopic sleeve gastrectomy with 12+ months of adjuvant multidisciplinary support

**DOI:** 10.1186/s12875-022-01629-7

**Published:** 2022-02-05

**Authors:** Prudence Carr, Tim Keighley, Peter Petocz, Michelle Blumfield, Graeme G. Rich, Felicity Cohen, Asha Soni, Isabella R. Maimone, Flavia Fayet-Moore, Elizabeth Isenring, Skye Marshall

**Affiliations:** 1 Department of Science, Nutrition Research Australia, Level 10, 20 Martin Place, Sydney, New South Wales; 2 Bariatric Gastroenterologist & Director, Bariatrics Australia, Sydney Adventist Hospital, Wahroonga, Australia; 3 Weightloss Solutions Australia, Varsity Lakes, Queensland Australia; 4 Nutrition Research Australia, Level 10, 20 Martin Place, Sydney, New South Wales; 5 Bond University Nutrition & Dietetics Research Group, Faculty of Health Sciences and Medicine, Bond University, Robina, Queensland Australia

**Keywords:** Obesity, Gastroplasty, Endoscopy, Laparoscopy, Bariatric surgery

## Abstract

**Background:**

The laparoscopic sleeve gastrectomy (LSG) and the incisionless endoscopic sleeve gastroplasty (ESG) weight loss procedures require further investigation of their efficacy, safety and patient-centered outcomes in the Australian setting.

**Methods:**

The aim was to examine the 6- and 12-month weight loss efficacy, safety, and weight-related quality of life (QoL) of adults with obesity who received the ESG or LSG bariatric procedure with 12+ months of adjuvant multidisciplinary pre- and postprocedural support. Data were from a two-arm prospective cohort study that followed patients from baseline to 12-months postprocedure from a medical center in Queensland. Percent excess weight loss (%EWL) was the primary outcome. Secondary outcomes were body composition (fat mass, fat-free mass, android:gynoid ratio, bone mineral content) via dual energy X-ray absorptiometry, weight-related QoL, lipid, glycemic, and hepatic biochemistry, and adverse events.

**Results:**

16 ESG (19% attrition; 81.2% female; aged:41.4 (SD: 10.4) years; BMI: 35.5 (SD: 5.2) kg/m^2^) and 45 LSG (9% attrition; 84.4% female; aged:40.4 (SD: 9.0) years; BMI: 40.7 (SD: 5.6) kg/m^2^) participants were recruited. At 12-months postprocedure, ESG %EWL was 57% (SD: 32%; *p* < 0.01) and LSG %EWL was 79% (SD: 24%; *p* < 0.001). ESG and LSG cohorts improved QoL (19.8% in ESG [*p* > 0.05]; 48.1% in LSG [*p* < 0.05]), liver function (AST: − 4.4 U/L in ESG [p < 0.05]; − 2.7 U/L in LSG [p < 0.05]), HbA1c (− 0.5% in ESG [p < 0.05]; − 0.1% in LSG [p < 0.05]) and triglycerides (− 0.6 mmol/L in ESG [p > 0.05]; − 0.4 mmol/L in LSG [*P* < 0.05]) at 12-months. Both cohorts reduced fat mass (p < 0.05). The ESG maintained but LSG decreased fat-free mass at 6-months (p < 0.05); and both cohorts lost fat-free mass at 12-months (p < 0.05). There were no adverse events directly related to the procedure. The ESG reported 25% mild-moderate adverse events possibly related to the procedure, and the LSG reported 27% mild-severe adverse events possibly related to the procedure.

**Conclusions:**

In this setting, the ESG and LSG were safe and effective weight loss treatments for obese adults alongside multidisciplinary support. Patients who elected the ESG maintained fat-free mass at 6-months but both cohorts lost fat-free mass at 12-months postprocedure. Patients who elected the LSG had large and significant improvements to weight-related quality of life. Further well-powered studies are required to confirm these findings.

**Trial registration:**

This study was registered prospectively at the Australia New Zealand Clinical Trials Registry on 06/03/2018, Registration Number ACTRN12618000337279.

**Supplementary Information:**

The online version contains supplementary material available at 10.1186/s12875-022-01629-7.

## Background

Much of the concern caused by the high global prevalence of obesity in adults (defined as BMI ≥30 kg/m^2^) is due to the metabolic consequences including the risk of type 2 diabetes mellitus, cardiovascular disease, obstructive sleep apnea, functional and psychosocial disability, poor overall quality of life (QoL), and mortality [1, 2]. Bariatric surgery, supported by adjuvant multidisciplinary lifestyle intervention, is the most effective treatment for obesity in terms of achieving and maintaining substantial weight loss as well as improving comorbidities [3, 4]; and the number of individuals who elect bariatric surgery is increasing in Australia and internationally [5–8].

Worldwide, 96% of bariatric surgeries are performed laparoscopically, with the laparoscopic sleeve gastrectomy (LSG) being the most common in Australia (71.5% of all procedures) [9, 10]. However, in line with general elective surgeries [11–14], bariatric surgeries like the LSG have adverse events (10–17%), postoperative mortality (0.3%) [15–19]. Despite being highly effective compared to other weight loss approaches [3, 4], the LSG has a failure rate of 15–50% and weight regain of 5% at 2-years to 70% at 6-years [15–19]. This has led to a demand for alternative effective weight loss procedures which may have fewer serious adverse events and/or have more accessible eligibility criteria [20]. Minimally-invasive endoscopic weight loss procedures have met this demand, such as the endoscopic sleeve gastroplasty (ESG), which is available at a lower BMI (e.g., ≥30 kg/m^2^ or lower with comorbidities) [21, 22].

A recent meta-analysis reported the incisionless ESG resulted in approximately 15% total body weight loss (TBWL) or 58% excess weight loss (EWL) at 6-months, which was sustained at 12-, 18- and 24-months [23], and evidence supports a low rate of serious adverse events (1–3%) [23–26]. Another meta-analysis reveled early TBWL following ESG was 9% at 1-month, and 12% at 3-months [27]. One study reported improvements in HbA1c, triglycerides, alanine aminotransferase (ALT), and systolic blood pressure at 12-months [25]. However, further research is required to confirm the efficacy and safety of the ESG, as well as measuring the underexplored outcomes of body composition and quality of life in both the ESG and LSG. Exploring these outcomes can provide insight into the expected outcomes of these procedures.

Therefore, the aim of this study was to examine the 6- and 12-month weight loss efficacy, safety, and weight-related quality of life (QoL) of adults with obesity who received the ESG or LSG bariatric procedure with 12+ months of adjuvant multidisciplinary pre- and postprocedural support. At study commencement, it was hypothesized that the ESG would demonstrate a weight loss effect and safety profile surpassing the criteria required to incorporate a new bariatric procedure into clinical practice as set by the American Society for Gastroenterology (> 25% EWL and < 5% risk of major complication at 12-months) (25). Further hypotheses were that improvements in comorbidity indicators and quality of life will be demonstrated with ESG and LSG patients, and that the ESG and LSG would have acceptable risk profiles in regard to gastrointestinal side-effects.

## Methods

A two-arm prospective cohort study was undertaken and reported according to the Strengthening the Reporting of Observational studies in Epidemiology (STROBE) checklist. This study was registered prospectively at the Australia New Zealand Clinical Trials Registry on 06/03/2018, Registration Number ACTRN12618000337279 [28] and received ethical approval from the Bond University Human Research Ethics Committee (HREC) (Reference: SM02936).

Eligible patients were consecutively recruited from a privately funded outpatient medical clinic (Weightloss Solutions Australia) in Queensland, Australia, which offered both ESG and LSG procedures. Adults aged ≥18 years who elected an ESG or LSG procedure from June 2018 to May 2019 were eligible and were recruited by written informed consent. Participants who lived > 1.5-h from the data collection site were ineligible to participate. Case selection for ESG and LSG occurred according to standard practice which involved a tailored discussion of the risks and benefits specific to the patient by the proceduralists (surgeons and/or gastroenterologists) who consult from the study site. Eligibility considerations for the procedures are outlined in Table S1; however, the ultimate decision was at the discretion of the physician and the patient.

### Study procedures

The ESG procedure was performed by one of the site proceduralists. Patients were admitted to a local hospital and placed under general anesthesia. A cap-based flexible endoscopic suturing system (OverStitch; Apollo Endosurgery, Austin, TX) was used to perform the procedure. The ESG was created by placing full thickness sutures from the incisura angularis to the proximal gastric body to mold the gastric lumen into a tubular configuration [21]. The number of sutures, bites, and suture pattern was extracted from the relevant hospital medical records when available.

The LSG procedure was performed as per standard practice by one of the site surgeons. Patients were admitted to a local hospital and placed under general anesthesia; local anesthetic was also administered at all local trocar entrance sites. The abdominal cavity was accessed via a supraumbilical incision and using a trocar. Two staplers were used commencing at the pylorus, up to the incisura angularis. The staple-line was inverted resulting in a gastric sleeve.

Prior to the procedure, all patients received a very low-calorie diet (VLCD) with duration triaged according to preprocedural BMI (1-week for participants with BMI < 35 kg/m^2^, 2-weeks for participants with BMI 35-49 kg/m^2^, and 3-weeks for participants with BMI > 50 kg/m^2^) due to its association with decreased procedure-related complications [29]. The VLCD was implemented using commercially available standardized meal replacements with the decision on which products or program used made by the patient and supported by the study site dietitians.

Following the procedure, standard follow up appointments with the multidisciplinary team, including a dietitian, a nurse, and a psychologist, were provided to all patients for 12-months (Table S2). Additional multidisciplinary appointments beyond the standard schedule were provided as needed. Following the procedure, a 6-week texture-modified diet was implemented for both ESG and LSG patients (Table S3), and dietary advice was given by the study site dietitian to maximize nutritional status and adherence. All patients were recommended to consume a daily multivitamin for life and were prescribed a proton pump inhibitor for the first month postprocedure which was continued thereafter as needed.

### Outcomes and data collection

The primary outcome measure was weight loss expressed as %EWL. Secondary outcome measures included %TBWL, BMI, QoL, body composition (fat mass, fat-free mass, android:gynoid fat mass ratio, bone mineral content), fasting serum biochemistry, gastrointestinal side-effects, and adverse events. Data were collected at baseline (> 5 days prior to procedure), 14-days postprocedure, 6-months postprocedure, and 12-months postprocedure. Adverse events were measured preprocedure, and postprocedure at 2-weeks, 6-months, and 12-months.

#### Weight and body composition

Weight and body composition were assessed at baseline, 6-months, and 12-month postprocedure. Weight, height, and body composition via dual energy X-ray absorptiometry (DXA) were measured by trained health professionals at Bond University Institute of Health and Sport (BIHS, Queensland, Australia) using calibrated scales (Wedderburn WM204), a wall mounted stadiometer with high-speed counter (Harpenden Model 602VR, Holtain Limited), and the Lunar Prodigy DXA, Encore Version 14.10.022 – GE Medical Systems Lunar [30], respectively. If patients did not attend the DXA scan at BIHS, weight and height was obtained from the study site medical records or was self-reported. BMI, %EWL (calculated on an unadjusted BMI of 25 kg/m^2^), and %TBWL were calculated from baseline body weight at 6- and 12-month postprocedure. Weight regain was defined as any increase in total body weight (kg) between 6- and 12-months. For those who experienced weight regain, the increase in total body weight was calculated as a percent change.

Fat mass (body material not identified as fat-free mass or bone mineral content), fat-free mass (body material identified as protein or water), and bone mineral content (amount of bone material) were measured in kilograms for the total body. Android fat mass (fat concentrated in the abdominal region), and gynoid fat mass (fat concentrated in the hips, upper thighs and buttocks) were used to calculate the android to gynoid fat mass ratio (%).

#### Weight-related quality of life

Weight-related QoL was measured via the Impact of Weight on Quality of Life (IWQOL-Lite) tool [31], which was self-completed by participants, at baseline and 6-months and 12-months postprocedure. The IWQOL-Lite is a 31-item validated questionnaire that measures five domains of QoL that affect obese individuals: physical function, self-esteem, sexual life, public distress, and work [31]. Each item reflects experiences in the past week from “never true” to “always true”, with higher scores indicating better quality of life. Scores were normalized to range from 0 (worst quality of life) to 100 (best quality of life).

#### Comorbidity indicators

Systolic and diastolic blood pressure (mmHg), fasting lipid profile (total cholesterol, LDL-cholesterol, HDL-cholesterol, triglycerides; mmol/L), fasting serum glucose (mmol/L), HbA1c (%), albumin (g/L), aspartate aminotransferase (AST, U/L) and ALT (U/L) were obtained from medical records at baseline, 6-months, and 12-months postprocedure. The hepatic steatosis index (HSI) was calculated using AST and ALT biochemistry, sex, BMI, and type 2 diabetes mellitus status; and was coded as ‘no non-alcoholic fatty liver disease (NAFLD) present’ using an HSI score < 30.0 or ‘risk of NAFLD’ using an HSI score > 36.0 [32]. The sensitivity and specificity of the HSI for detecting NAFLD with these cut-off scores has been reported as 93% and 92%, respectively [32].

#### Gastrointestinal side-effects and adverse events

Gastrointestinal symptoms and side-effects were evaluated by the Gastrointestinal Symptom Rating Scale (GSRS) which was self-completed by participants at baseline, 6-months, and 12-months postprocedure. The GSRS is a 15-item questionnaire, which asks participants to rate symptoms experienced in the past week on a Likert scale of 1 (no symptom) to 7 (severe/frequent symptom) [33]. Domains include abdominal pain, reflux, diarrhea, indigestion, and constipation.  Scores were normalized to 0 (no symtpoms) to 100 (worst symptoms).

Non-gastrointestinal symptom adverse events were classified according to the National Institute of Health in terms of severity (mild, moderate, severe), expectedness (unexpected, expected), and relatedness to the procedure (definitely related, possibly related, not related) [34]. The etiology of directly- or possibly-related events and their treatment was recorded. Events which were not-related (e.g., influenza, urinary tract infection, melanoma) were not described in this study.

### Statistical analysis

Minimum sample sizes to detect a significant %EWL were small due to the large effect size. With 90% power and 0.05α error probability, each procedure required a sample size of approximately six persons as calculated by G* Power. However, to effectively evaluate adverse events and to increase the power of the study, all eligible participants within the recruitment period were invited to participate.

Descriptive statistics were reported as means (standard deviation, SD) or counts and percentages (%). Paired t-tests, 2-sided, were performed between baseline versus 6-months and baseline versus 12-months to measure change overtime within each procedural cohort. Paired t-tests were chosen in preference to repeated measures analysis due to the higher attrition over time and in inadvisability of imputation for missing data in this context. As the paired t-test was chosen, any participant with missing follow-up data was excluded from the analyses for that variable, therefore, an ‘intention-to-treat’ approach was not utilized. Pearson’s and Spearman’s correlation coefficients were generated to understand the relationship between quality of life and %EWL. Normality assumptions were checked by inspection of Q-Q (quantile-quantile) plots, and by confirming the results of t-tests using non-parametric alternatives (Wilcoxon matched-pairs tests). *P*-values were adjusted using the false discovery rate method. McNemar’s tests were used to check for changes in proportions of abnormal biochemistry values between time points. As participants were not randomized but were observed as cohorts, outcomes were not directly compared between procedures. Most analyses were performed using the R software version 4.0.3 (R Foundation for Statistical Computing, Vienna, Austria), and a minority using SPSS version 27 (IBM, New York). Values of *p* < 0.05 were regarded as indicating statistical significance.

## Results

**Fig. 1:**
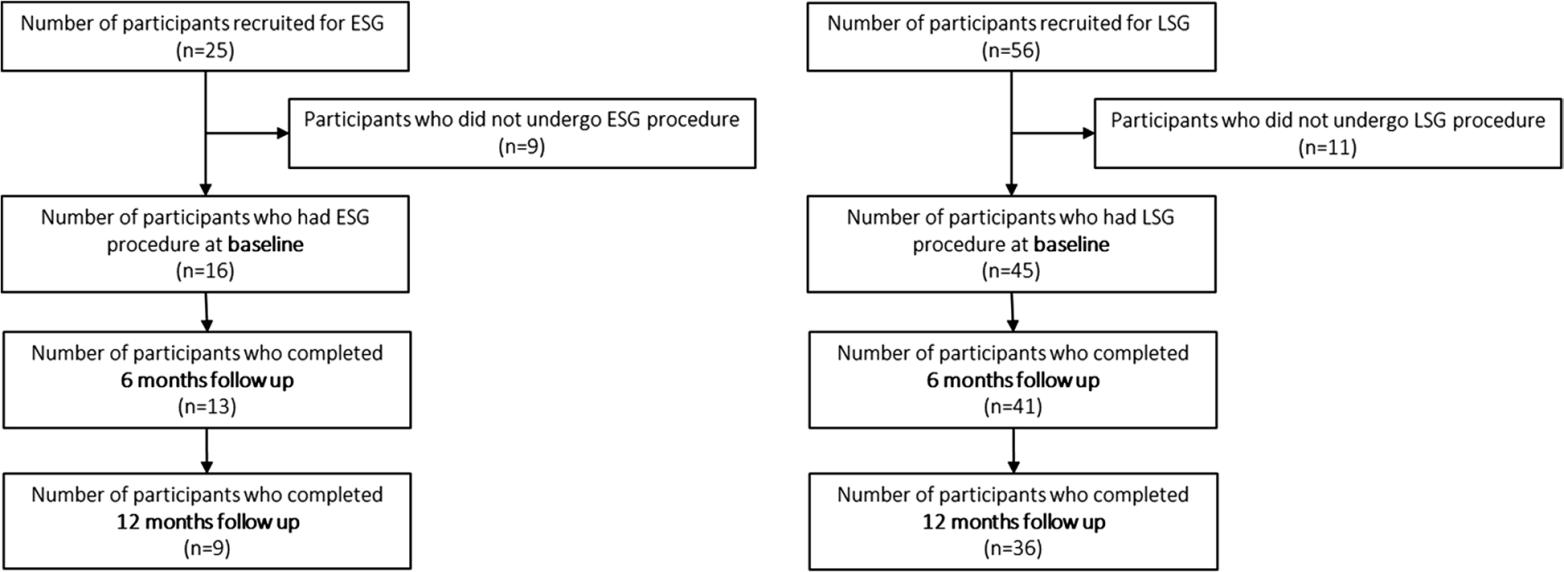
Recruitment, withdrawals and study visit attendance of the ENvISaGE study participants undergoing either ESG or LSG procedure

The 16 recruited ESG participants who attended their procedure were 81.2% female, aged 41.4 (SD: 10.4) years, with a BMI of 35.5 (SD: 5.2) kg/m^2^ (range: 29.9 to 45.1 kg/m^2^). The 45 recruited LSG participants who attended their procedure were 84.4% female; aged 40.4 (SD: 9.0) years, with a BMI 40.7 (SD: 5.6) kg/m^2^ (range: 29.9 to 51.5 kg/m^2^) **(**Table 1**;** Fig. 1**)**.

**Table 1:** Baseline characteristics of the study participants, by procedure

Patient characteristic	ESG ***N*** = 16	LSG ***N*** = 45
Age, mean (SD), years	41.4 (10.4)	40.4 (9.0)
Sex, n (%) female	13 (81.2)	38 (84.4)
Ethnicity, n (%)		
Caucasian	12 (75)	40 (89)
Asian	2 (12)	0 (0)
African	0 (0)	1 (2)
Other	1 (6)	0 (0)
Not-disclosed	1 (6)	2 (4)
Area of residence, n (%)		
Metropolitan	14 (88)	44 (98)
Rural	2 (12)	1 (2)
BMI, kg/m^2^, mean	35.5 (5.2)	40.7 (5.6)
BMI < 35, n (%)	9 (56.3)	6 (13.3)
BMI 35- < 50, n (%)	7 (43.8)	37 (82.2)
BMI ≥50, n (%)	0 (0)	2 (4.4)
Body composition^a^, mean (SD)		
Fat mass, kg	48.5 (8.7)	57.9 (13.3)
Fat-free mass, kg	49.7 (9.9)	53.1 (9.7)
Bone mineral content, kg	2.8 (0.3)	2.8 (0.5)
SBP (mmHg)^b^	129.3 (12.4)	123.3 (15.1)
DBP (mmHg)^b^	87.6 (9.2)	83.2 (10.2)
Type 2 Diabetes Mellitus, n (%)	0 (0)	2 (4)
Hypertension, n (%)	7 (44)	14 (31)
Dyslipidemia, n (%)	3 (19)	7 (16)
Obstructive sleep apnea, n (%)	0 (0)	6 (13)
Osteoarthritis/joint pain, n (%)	8 (50)	33 (73)
Non-alcoholic fatty liver disease, n (%)	1 (6)	3 (7)
Polycystic ovary syndrome (females only), n (%)	1 (6)	8 (18)
Gastroesophageal reflux disease, n (%)	4 (25)	17 (38)
Depression or anxiety, n (%)	7 (44)	17 (38)
Gestational diabetes mellitus (females only), n (%)	2 (12)	6 (13)
Back pain, n (%)	10 (62)	31 (69)
Asthma, n (%)	3 (19)	16 (36)
HSI score^c^, mean (SD)	46.8 (6.3)	52.8 (6.7)
Risk of NAFLD^d^	15 (94)	44 (100)

^a^Body composition: 6 participants did not attend DXA scan (ESG), 11 participants did not attend DXA scan (LSG)

^b^2 participants missing information (LSG)

^c^1 participant missing information (LSG)

^d^1 participant (ESG) indeterminate; 1 participant missing information (LSG)

*Abbreviations*: *BMI* body mass index, *SBP* systolic blood pressure, *DBP* diastolic blood pressure, *HSI* hepatic steatosis index, *NAFLD* non-alcoholic fatty liver disease, *ESG* endoscopic sleeve gastroplasty, *LSG* laparoscopic sleeve gastrectomy

The mean time of follow-up for the 6- and 12-month postprocedural timepoints were 7.4-months and 13.6-months for weight; 6.6-months and 12.5-months for biochemistry; 8.1-months and 14.7-months for GSRS and QoL; and 7.4-months and 13.7-months for DXA body composition, respectively. ESG procedure characteristics (numbers of sutures and bites) were inconsistently provided by the hospitals and therefore not analyzed nor reported.

Baseline characteristics of participants who were recruited but did not complete the procedure are presented in Table S4. There was 19% and 43% attrition in the ESG cohort at 6- and 12-months postprocedure, respectively; and 9% and 20% attrition in the LSG group at 6- and 12-months postprocedure, respectively. The higher attrition in the ESG group was due to COVID-19, as the majority of the ESG cohort were recruited later than the LSG. The COVID-19 pandemic and corresponding government restrictions meant many participants could not attend their 12-month follow-up visits which were scheduled to occur in 2020, leading to increased overall attrition (44% ESG; 20% LSG) and high non-attendance at DXA scans (60% ESG; 26% LSG).

### Weight loss

Where total body weight was available, 31% (38% ESG; 29% LSG) were obtained via medical records as opposed to measured by the researchers at baseline, 22% (36% ESG; 17% LSG) at 6-months postprocedure, and 31% (56% ESG; 25% LSG) at 12-months postprocedure. The ESG and LSG cohorts both reported reductions in %EWL and %TBWL from baseline to 6-months postprocedure and from baseline to 12-months postprocedure **(**Table 2**)**. %EWL in the ESG cohort was 51% (SD: 11%; *p* < 0.001) at 6-months and 57% (SD: 32%; *p* < 0.01) at 12-months postprocedure; where 78% achieved > 25% EWL at 12-months. In the LSG cohort, %EWL was 66% (SD: 25%; p < 0.001) at 6-months and 79% (SD: 24%; p < 0.001) at 12-months postprocedure; and 97.2% achieved > 25% EWL at 12-months **(**Table 2**)**.

**Table 2:** Body mass index, excess weight loss, and percent total body weight loss at 6-months follow-up for study participants undergoing either ESG or SG procedure

	BMI		EWL (%)		Reinhold Classification				TBWL (%)		TBWL < 20%	TBWL ≥ 20%
					EWL < 25%	EWL 25–49.9%	EWL 50–74.9%	EWL ≥ 75%				
	n	Mean (SD)	n	Mean (SD)	n (%)	n (%)	n (%)	n (%)	n	Mean (SD)	n (%)	n (%)
**ESG**												
6-month follow-up	13	30.5 (2.9)***	13	51 (11)***	0 (0)	6 (46.2)	7 (53.8)	0 (0)	13	15 (6)***	11 (84.6)	2 (15.4)
12-month follow-up	9	29.9 (3.6)**	9	57 (32)**	2 (22.2)	2 (22.2)	2 (22.2)	3 (33.3)	9	18 (11)**	4 (44.4)	5 (55.6)
**LSG**												
6-month follow-up	41	31.4 (5.1)***	41	66 (25)***	0 (0)	11 (26.8)	17 (41.5)	13 (31.7)	41	24 (6)***	11 (26.8)	30 (73.2)
12-month follow-up	36	29.2 (5.1)***	36	79 (24)***	1 (2.8)	3 (8.3)	12 (33.3)	20 (55.6)	36	30 (8)***	4 (11.1)	32 (88.9)

**P < 0.01 for the difference between baseline and 12-months follow-up

***P < 0.001 for the difference between baseline and 6-months follow-up

*Abbreviations*: *ESG* endoscopic sleeve gastroplasty, *EWL* excess weight loss, *LSG* laparoscopic sleeve gastrectomy, *TBWL* total body weight loss

In the ESG cohort, %TBWL was 15% (SD: 6%; *p* < 0.001) at 6-months and 18% (SD: 11%; p < 0.01) at 12-months; while the LSG cohort achieved 24% (SD: 6%; p < 0.001) TBWL at 6-months and 30% (SD: 8%; p < 0.001) at 12-months postprocedure **(**Table 2**)**.

The mean BMI for both cohorts was reduced from the “obese” (BMI > 30.0 kg/m^2^) range to the “overweight” (BMI 25–29.9 kg/m^2^) range (*p* < 0.05) **(**Table 2**)**. Between 6- and 12-months, 30% of ESG and 6% of LSG participants experienced weight regain (5.1% [SD: 3.1%] weight increase for ESG and 3.2% [SD: 2.5%] weight increase for LSG]).

### Body composition

Both cohorts achieved reductions in fat mass from baseline to 6-months (ESG: − 10.3 kg [SD: 4.9 kg], *p* = 0.011; LSG: − 21.5 kg [SD: 7.2 kg], p < 0.001) and the LSG cohort achieved fat mass reductions at 12-months (− 26.0 kg [SD: 10.7 kg], *p* < 0.05) (Table S5)**.** Fat-free mass was observed to significantly increase from baseline to 6-months postprocedure in the ESG cohort (49.7 kg [SD: 9.9 kg] to 50.0 kg [SD: 12.8 kg]; p < 0.05); however, this then significantly decreased at 12-months (46.7 kg [SD: 2.7 kg], p < 0.05). Earlier and larger decreases in fat-free mass were observed in the LSG cohort from baseline to 6-months [53.1 kg [SD: 9.7 kg] to 47.5 kg [SD: 8.4 kg], p < 0.05) and 12-months (48.3 kg [SD: 9.2 kg] p < 0.05; Table S5).

The android:gynoid fat mass ratio showed similar improvements in both procedures at 6- (ESG: -0.1 [SD: 0.1]; LSG: -0.1 [SD: 0.1]) and 12-months postprocedure (ESG: -0.1 [SD: 0.1]; LSG: -0.1 [SD: 0.1]); however, only the LSG was significant (p < 0.05).

No difference in bone mineral content was found in the ESG cohort (*p* = 0.358). Although the LSG cohort achieved significant bone mineral content change from baseline to 6- and 12-month postprocedure (2.8 (SD: 0.5) kg; *p* = 0.003), the significant *p*-value was generated by a decrease in a single participant; and the mean bone mineral content for the cohort remained stable (Table S5).

### Weight-related quality of life

**Fig. 2:**
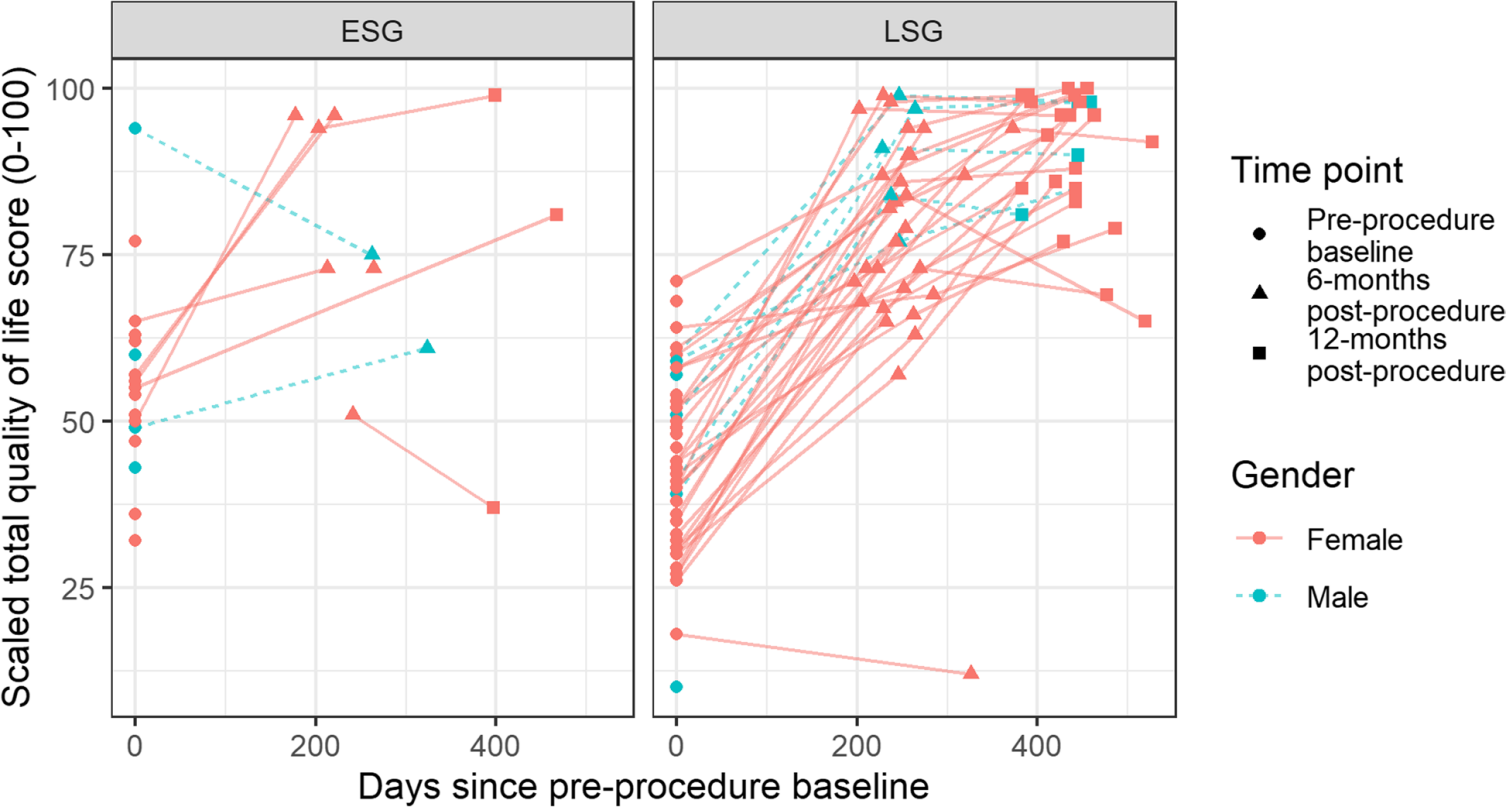
Weight related quality of life score of study participants undergoing either ESG or LSG procedure at baseline, 6-month follow-up and 12-month follow-up

Individual changes in weight-related QoL by cohort and sex are shown in Fig. 2 and by QoL domain in Fig. S1. The ESG cohort improved QoL from 58.0 (SD: 14.6) to 78.2 (SD: 15.9) at 6-months which was maintained at 12-months (77.8%; SD: 28.2), and the LSG from 41.5 (SD: 14.2) to 79.3 (SD: 16.8) at 6-months, which was further improved to 89.6 (SD: 9.7) at 12-months. However, only the improvements in LSG cohort were statistically significant (*p* < 0.001; Table S5). The improvements to QoL in both procedures were represented by improvements across all QoL domains; however, the greatest changes were in self-esteem, physical function, and sexual life.

QoL total scores were moderately positively correlated with %EWL for the ESG (*r*: 0.43 at 6-months [*p* = 0.247]; *r*: 0.60 at 12-months [*p* = 0.400]) and LSG cohorts (*r*: 0.47 at 6-months [*p* = 0.005]; *r*: 0.43 at 12-months [*p* = 0.025]); however, only the LSG correlations were statistically significant.

### Fasting serum biochemistry

All mean glycemic measures, lipid profiles, albumin, and liver enzymes were in the normal ranges at all timepoints in both cohorts (Table S5); except for the total cholesterol which was borderline elevated (> 5.5 mmol/L) in the ESG group at baseline. HbA1c in the ESG cohort significantly decreased from baseline to 12-months (5.0% [SD: 0.3%] to 4.7% [SD: 0.3%], *p* < 0.05) postprocedure (Table S5). Significant reductions in glucose and HbA1c were observed in the LSG cohort from baseline to 6-months (5.4 [SD: 1.2] to 4.8 [SD: 0.5] mmol/L, p < 0.05; and 5.3% [SD: 0.5%] to 5.1% [SD: 0.5%], p < 0.05, respectively) and to 12-months (5.4 [SD: 1.2] to 5.0 [SD: 0.5] mmol/L, p < 0.05; and 5.3% [SD: 0.5%] to 5.2% [SD: 0.7%], p < 0.05, respectively) postprocedure.

No differences in the mean values were found in either cohort for total cholesterol, LDL cholesterol, HDL cholesterol, nor triglycerides at 6-months postprocedure (*p* > 0.05 for all; Table S5). However, the LSG cohort observed significant improvements in HDL cholesterol and triglycerides from baseline to 12-months postprocedure. Liver function improved in the LSG cohort at 6- and 12-months postprocedure (*p* < 0.001 for all), while HSI score improved in both cohorts at 6-months (ESG *p* = 0.024; LSG p < 0.001; Table S5), which was maintained at 12-months.

When categorised, both cohorts had low rates of abnormal biochemistry values (Table S5). Total cholesterol was the most frequent abnormal biochemistry value for both cohorts at baseline (ESG: 44%; LSG: 31%). Low HDL cholesterol was the most common abnormal value at 6- and 12-months postprocedure for the ESG cohort; but high total cholesterol remained the most common abnormal value at 6- and 12-months postprocedure for the LSG cohort. Due to small sample sizes and low rates of abnormal values; most comparator tests to detect change in proportions over time were underpowered or could not be conducted. However, it was found that significantly fewer LSG participants had elevated ALT at 6-months postprocedure compared to baseline (Table S5).

### Adverse events and gastrointestinal side-effects

No changes in gastrointestinal symptom (GSRS) scores were found from baseline to 6- and 12-months postprocedure (ESG: *p* = 0.454; LSG: *p* = 0.754; Table S5)**.** There were no non-gastrointestinal symptom-related adverse events which were directly related to the procedure. The ESG cohort reported four (25%) mild-moderate possibly-related adverse events; and the LSG cohort reported 12 (27%) mild, moderate, or severe possibly-related adverse events (Table S6).

## Discussion

This study demonstrated the safety and efficacy of both ESG and LSG with 12+ months of adjuvant multidisciplinary pre- and postprocedural support for the treatment of obesity, and for the first-time, assessed changes in body composition and QoL using validated tools in ESG patients. Although the LSG is the most prevalent bariatric surgery worldwide and has been highly researched, this study also contributes the first published DXA body composition data for the LSG outside of China [35–38] or New Zealand [39].

Patients in both procedural cohorts achieved reductions in BMI, %EWL, %TBWL, and glycemic markers from baseline to 6-months and/or 12-months postprocedure. However, due to underpowered analyses in the ESG cohort, only patients in the LSG cohort reported statistically significant improvements in QoL, lipid profiles, and liver function biomarkers. Although biomarker improvements were achieved in both cohorts, it should be acknowledged baseline sample means for all measures were within the recommended ranges. The characteristics of ESG and LSG cohorts were heterogenous, but both had no to very low rates of type 2 diabetes and cardiovascular disease. Further research is required to examine the effects on glycemic and lipid measures in cohorts with established disease at baseline.

The small ESG sample size and high attrition rates, particularly at 12-months, led to underpowered analyses and prevent generalization. While results therefore need to be interpreted with caution, findings suggest the ESG cohort far exceeded the thresholds set by the American Society for Gastroenterology for defining a valuable endoscopic procedure for weight loss which is > 25% EWL and < 5% risk of major complication at 12 months [40]. As the ESG is a relatively new procedure and weight regain is common in obesity treatment, further data on its long-term effectiveness are required [41], with the Mayo Clinics’ MERIT trial results due shortly [42]. However, recent meta-analyses have confirmed procedural safety [23, 27, 43].

The weight loss reported in both ESG and LSG cohorts was consistent with published literature. A recent meta-analysis directly compared ESG and LSG [43], finding the ESG %TBWL at 6-months was approximately 15%, and LSG %TBWL was approximately 30% at 12-months [43], thereby directly aligning with the findings from the current Australian cohorts. Previous evidence suggests that bariatric surgeries such as the LSG have a 6% rate of weight regain at 2-years which was the rate reported by the current study at 12-months [15]. The regain rate for the ESG in the current study was similar to international literature, where Abu Dayyeh et al. [44] reported a slightly higher rate of 37.5% but at a later timepoint (20-months). Due to the observational nature of this study, the difference in regain rates reported by ESG and LSG cohorts may be only partially explained by the procedure; but may also be due to other factors such as differences in baseline characteristics. Although regain rates in this study were similar to those reported in international literature, the percent of weight regained was minimal (≤5.1%) and should be interpreted in the context of patients having intensive multidisciplinary support. The weight regain rates and amounts regained are also likely to be biased by the high attrition which occurred at 12-months. Whilst well understood in LSG patients [45, 46], future research should explore and confirm the rate, etiological factors, and characteristics of weight recidivism in ESG patients.

The retention and/or gain of fat-free mass, which largely reflects skeletal muscle, following bariatric surgery is important to maintain metabolism, support weight maintenance, and prevent complications such as sarcopenia [47–49]. Despite a loss of fat-free mass being expected following bariatric surgery [49] and observed early in the LSG cohort, the ESG cohort managed to increase fat-free mass at 6-months. Although the increase of approximately 0.3 kg was statistically significant, it is not clinically significant, so should be interpreted as maintaining fat-free mass only. Whilst only representing a small cohort of patients, this finding suggests the slower but still significant weight loss in the ESG cohort may prevent loss of fat-free mass; but resistance exercise is equally important for both cohorts to maintain fat-free mass at 12-months [50]. The significant and early loss of fat-free mass in the LSG cohort also suggests that the greater %EWL and %TWBL achieved for the LSG cohort compared to the ESG cohort is partially explained by higher loss of fat-free mass. Although maintaining fat-free mass would decrease calculated %EWL and %TBWL, intensive multidisciplinary support to assist patients in participating in suitable resistance exercise to build and maintain fat-free mass has been demonstrated to lead to higher long term weight loss [50]. Despite clear differences in fat mass and lean mass changes between ESG and LSG procedures, both cohorts reported the same improvement of − 0.1 in the android:gynoid fat mass ratio at 6- and 12-months follow-up timepoints. As this android:gynoid fat mass ratio is highly associated with chronic disease risk [51, 52], the long term impacts of both procedures on disease incidence is of interest, and is currently unknown for the ESG due to its recency. As the LSG cohort had higher mean preoperative fat mass; procedural %EWL rather than %TBWL should be referred to when making decisions for procedure selection and referral.

This study observed maintenance of bone mineral content in both cohorts which is a positive finding; however, bone mineral density was not measured. Good evidence exists to show that bariatric surgery results in significant bone mineral density loss [53]; however, it remains unknown if this occurs in patients who elect the ESG. The ESG procedure has no mal- or hypoabsorptive mechanisms; and therefore, its effect on bone mineral density requires examination as this is an important consideration for clinical and patient decision making as well as follow-up care.

Patients with morbid obesity are shown to have lower QoL compared with the general population and both endoscopic and surgical bariatric procedures are associated with improved QoL [31, 54–56]. The lack of a statistically significant improvement in QoL reported in the ESG cohort was related to this measure being underpowered due to a small sample. Despite no statistical improvement, overall QoL and all domains of QoL in the ESG group were clinically meaningful and further research with large sample sizes is needed to confirm the finding. Although causation cannot be inferred, the moderate correlations between QoL and %EWL suggests there may be many factors influencing QoL improvements following bariatric procedures, a finding which aligns with lifestyle obesity treatments [57]. In depth qualitative research has revealed that weight loss following bariatric surgery has led to complex changes in self-perception as well as impacting upon relationships, which are closely aligned with mental health and quality of life [58]. Further, research following endoscopic bariatric procedures found that quality of life was enhanced alongside improvements to mental health which occurred when patients were provided with postoperative multidisciplinary support including psychology [56].

This study was limited by a small sample size in the ESG cohort and high attrition rates across the 12-months for both cohorts, especially for DXA body composition and measured total body weight, which is representative of the impacts of COVID19, and the practice context where ESG is emerging and attrition is common [59]. As this study analyzed multiple outcome variables to answer the research question; there is a chance of type I errors. However, due to the small sample size, there is a higher chance of type II errors, particularly for the ESG cohort. Interpretation of the effects of ESG were also limited by lack of suture data available. Despite high 12-month attrition, attendance at 6-months for the primary study outcomes was high (ESG 81%; LSG 91%). This study was conducted in a center specialized in performing bariatric surgery, with 12-months of compulsory adjuvant multidisciplinary follow-up, therefore the generalizability of outcomes may be limited. Finally, there is risk of measurement bias from the inclusion of weight data from patient medical records when participants did not attend BIHS for data collection.

As this study was limited by its observational nature and patients were not randomized to procedures, no conclusions about causality can be drawn, nor a direct comparison of ESG and LSG procedures. Instead, this study provides novel data to describe the Australian bariatric practice setting from which the multidisciplinary team may consider clinical applications and interpretations.

## Conclusions

In adults with obesity sampled in Queensland, Australia,  elected ESG or LSG procedures with 12+ months of adjuvant pre- and postprocedural multidisciplinary support were safe and effective treatments at 6- and 12-months postprocedure. The LSG cohort demonstrated improvements in quality of life, glycemia, liver function, and lipid profiles, whereas the ESG cohort demonstrated maintenance of fat-free mass 6-months postprocedure and improved glycemia at 12-months postprocedure. Larger studies are needed to confirm these findings and evaluate the long-term effectiveness of the ESG and LSG procedures as well as measure the effect of the ESG on bone mineral density and quality of life.

## Supplementary Information

**Additional file 1: Table S1.** Eligibility considerations of proceduralists who provide ESG and LSG. **Table S2**. Standard follow-up appointments with the multidisciplinary team for patients who have had an ESG or LSG. **Table S3.** Post-procedure texture modified diet plan for ESG and LSG patients. **Table S4.** Baseline characteristics of participants who did not have the procedure. **Table S5**. Gastrointestinal symptoms, quality of life, body composition, and pathology measures of ESG and LSG participants at baseline, 6-month, and 12-month follow-up. **Table S6**. Non-gastrointestinal symptom-related adverse event etiology, severity, expectedness, relatedness, and treatment which occurred in adults who elected ESG and LSG procedures from day of surgery to 12-months post-procedure. **Fig. S1.** Weight related quality of life score by domain of study participants at baseline, 6-months, and 12-months follow-up undergoing either ESG or LSG procedure.

## Data Availability

The datasets used and/or analysed during the current study are available from the corresponding author on reasonable request.
