# Supplementary material for: Efficacy and safety of endoscopic sleeve gastroplasty and laparoscopic sleeve gastrectomy with 12+ months of adjuvant multidisciplinary support
Source: BMC Prim Care. 2022 Feb 5;23:26. doi: 10.1186/s12875-022-01629-7 (PMC8817771; doi:10.1186/s12875-022-01629-7)
Supplement: Supplementary file 1 — Additional file 1: Table S1. Eligibility considerations of proceduralists who provide ESG and LSG. Table S2. Standard follow-up appointments with the multidisciplinary team for patients who have had an ESG or LSG. Table S3. Post-procedure texture modified diet plan for ESG and LSG patients. Table S4. Baseline characteristics of participants who did not have the procedure. Table S5. Gastrointestinal symptoms, quality of life, body composition, and pathology measures of ESG and LSG participants at baseline, 6-month, and 12-month follow-up. Table S6. Non-gastrointestinal symptom-related adverse event etiology, severity, expectedness, relatedness, and treatment which occurred in adults who elected ESG and LSG procedures from day of surgery to 12-months post-procedure. Fig. S1. Weight related quality of life score by domain of study participants at baseline, 6-months, and 12-months follow-up undergoing either ESG or LSG procedure. [file 12875_2022_1629_MOESM1_ESM.docx]

**Table S1:** Eligibility considerations of proceduralists who provide ESG and LSG

| Physiological eligibility criteria | Contraindications for ESG and LSG |
| --- | --- |
| - Excessive adiposity posing a long-term health risk indicated by a BMI in the obese categories (BMI >30kg/m2). - Excessive adiposity posing a long-term health risk indicated by a BMI in the overweight category (e.g., >26kg/m2) if there are existing comorbidities such as hypertension, type 2 diabetes mellitus or cardiovascular disease. | - Liver cirrhosis particularly if associated with portal hypertension, coagulopathy or thrombocytopenia. - A medical comorbidity not allowing general anesthesia. - Current pregnancy or planned pregnancy within 12-18 months. Future pregnancy would need to be discussed on a case-by-case basis as no guidelines for bariatric endoscopic procedures exist. The Obesity Society (TOS) and American Society for Metabolic and Bariatric Surgery (ASMBS) recommend avoiding pregnancy for 12 to 18 months following a bariatric surgery.^[[1]](#endnote-1)^ - Psychiatric disorders that would interfere with weight loss or lead to patient safety concerns. - Anatomic variation precluding safe progression to ESG - Mucosal bleeding lesions such as ulceration, - Neoplastic or pre-malignant lesions, - Large hiatal hernias (>3cm) or - Evidence of portal hypertension such as endoluminal varices or portal hypertensive gastropathy. |

**Table S2**. Standard follow-up appointments with the multidisciplinary team for patients who have had an ESG or LSG

| **Health-professional** | **Method** | **Time-point** |
| --- | --- | --- |
| Accredited Practicing Dietitian (APD) | Mode: in-person or telephone  Duration: 15 minutes | 2-weeks, 4-weeks, 6-weeks, 3-months, 6-months, 9-months, 12-months post-procedure; additional follow-up as needed. |
| Registered Nurse | Mode: in person or telephone  Duration: 15mins | Day-3, 7 and 14 post-procedure; additional follow-up as needed. |
| Psychologist | Mode: in-person or telephone  Duration: 30 minutes | As needed or requested. |

**Table S3.** Post-procedure texture modified diet plan for ESG and LSG patients

| **Diet type and purpose** | **Description** | **Implementation** |
| --- | --- | --- |
| Clear fluids: aims to replace or maintain the hydration and allow for minimum residue in the intestinal tract. Nutritionally inadequate. | Composition: Only fluids or foods that are liquid at room temperature ^a^. Typically includes fat-free clear soups, juice, jelly, cordial, and water. Black tea and coffee are usually excluded.  Amount: Sipping throughout the day, with a goal of 2.5L/day | During hospital admission (variable length) ^b^ |
| Full fluids: aims to provide oral liquids to maintain hydration, reduce stomach distension, and leave minimum residue in the intestinal tract post-procedure. Nutritionally inadequate. | Composition: Only fluids or foods that are liquid at room temperature ^a^. Typically includes fat-free clear soups, juice, milk, water, cordial, jelly, smoothies, high-protein oral nutrition supplement. Black tea, coffee and carbonated drinks are excluded.  Amount: Sipping throughout the day, with a goal of 2.5L/day | Day of discharge to day 13 post-procedure. |
| Full fluids plus puree foods: to provide food which is smooth, requires no chewing, and aims to be nutritionally adequate. No fluids consumed 30 minutes before or after food. | Composition: Commercial food which has the consistency of baby oatmeal or pudding; or home-made food which is pureed to a smooth consistency and can be easily cut with the side of a fork. Food should be cohesive enough to hold its shape on a spoon or if molded.  Nutritional composition: goal of 50% protein, 30% low-carbohydrate vegetables, 20% wholegrains or legumes.  Amount: Commence with 4-5tsp of puree food per meal and aim for 0.5 per meal by day 27; with 5-6 meals per day. Goal of 2.5L fluid per day. | Day 14 to 27 post-procedure. |
| Soft foods: to provide food which can be easily chewed and nutritionally adequate, as a transition to a normal diet. No fluid-type restrictions, however avoid fluid consumed 30 minutes before or after food. | Composition: Includes foods which may be naturally soft or are cooked or altered to obtain a soft texture. Foods should be moist, easily crumbled, or served with sauce to increase the moisture content. Typically includes soft meat, fish, chicken, cereals, cooked vegetables, canned or fresh fruits. Skins, seeds, and bread are avoided.  Nutritional composition: goal of 50% protein, 30% low-carbohydrate vegetables, 20% wholegrains or legumes.  Amount: 0.5-1C per meal; with 5-6 meals per day. Goal of 2.5L fluid sipped throughout the day. | Day 28 to day 42 post-procedure. |
| Normal diet: to consume a wide variety of foods which are nutritionally adequate. No fluid-type restrictions. No fluid-type restrictions, however avoid fluid consumed 30 minutes before or after food. | Composition: Includes the full range of available foods, with guidance on food choice / meal plans to achieve a healthy dietary pattern.  Nutritional composition: goal of 50% protein, 30% low-carbohydrate vegetables, 20% wholegrains or legumes.  Amount: 0.5-1C per meal; with 5-6 meals per day. After 6 months post-procedure ^c^, aim for 1C per main meal (3/day) and 0.5C per snack (2/day). Goal of 2.5L fluid sipped throughout the day. | Day 43 post-procedure onwards. |

C, cup; L, liter

1. Nutritional content of foods is modified based on patient symptoms. For example, if the patient is experiencing reflux, fat-free fluid options are used; if the patient is experiencing diarrhea or dumping syndrome, sugar-free fluid options are used.
2. If patients have an unplanned extended length of stay in hospital progression to the next diet steps may occur during the hospital admission.
3. Patients may be able to achieve 1C portions of foods from variable timepoints, usually ranging from two weeks post-procedure to 18-months procedure.

**Table S4.** Baseline characteristics of participants who did not have the procedure

| **Patient characteristic** | **ESG**  **N=9** | **LSG**  **N=11** |
| --- | --- | --- |
| Age, mean (SD), years | 45.3 (8.7) | 39.0 (8.3) |
| Sex, n (%) female | 7 (77.8) | 10 (90.9) |
| Ethnicity, n (%) |  |  |
| Caucasian | 9 (100) | 10 (90.9) |
| Asian | 0 (0) | 0 (0) |
| African | 0 (0) | 0 (0) |
| Indigenous Australian | 0 (0) | 0 (0) |
| Pacific Islander | 0 (0) | 1 (9.1) |
| Other | 0 (0) | 0 (0) |
| Not-disclosed | 0 (0) | 0 (0) |
| Area of residence, n (%) |  |  |
| Metropolitan | 8 (88.9) | 10 (90.9) |
| Rural | 1 (11.1) | 1 (9.1) |
| BMI, kg/m^2^, mean | 32.8 (2.6) | 40.8 (8.4) |
| BMI <35, n (%) | 7 (77.8) | 2 (18.2) |
| BMI 35-<50, n (%) | 2 (22.2) | 8 (72.7) |
| BMI ≥50, n (%) | 0 (0) | 1 (9.1) |
| Body composition^1^, mean (SD) |  |  |
| Fat mass, kg | 38.9 (2.3) | 59.4 (11.7) |
| Fat-free mass, kg | 40.8 (3.8) | 51.2 (2.4) |
| Bone mineral content, kg | 2.4 (0.1) | 2.8 (0.2) |
| SBP (mmHg) ^2^ | 125.9 (10.8) | 124.3 (11) |
| DBP (mmHg) ^2^ | 79.0 (9.1) | 85.2 (8.1) |
| Type 2 Diabetes Mellitus, n (%) | (0) | 0 (0) |
| Hypertension, n (%) | 2 (22.2) | 2 (18.2) |
| Dyslipidemia, n (%) | 4 (44.4) | 2 (18.2) |
| Obstructive sleep apnea, n (%) | 0 (0) | 1 (9.1) |
| Osteoarthritis/joint pain, n (%) | 3 (33.3) | 8 (72.7) |
| Non-alcoholic fatty liver disease, n (%) | 1 (11.1) | 1 (9.1) |
| Polycystic ovary syndrome (female only), n (%) | 1 (11.1) | 1(9.1) |
| Gastroesophageal reflux disease, n (%) | 5 (55.5) | 5 (45.4) |
| Depression or anxiety, n (%) | 5 (55.5) | 6 (54.5) |
| Gestational diabetes mellitus (females only), n (%) | 2 (22.2) | 3 (27.3) |
| Impaired fasting glucose, n (%) | 0 (0) | 1 (9.1) |
| Back pain, n (%) | 5 (55.5) | 10 (90.9) |
| Asthma, n (%) | 3 (33.3) | 3 (27.3) |
| HSI score^3^, mean (SD) | 39.8 (1.3) | 50.9 (2.9) |
| NAFLD, n (%) | 2 (22.2) | 5 (45.4) |

^1^ Body composition, 7 did not attend DXA (ESG), 8 did not attend DXA (LSG)
^2^ 3 participants did not attend (LSG)
^3^ 7 participants missing information (ESG), 6 participants missing information (LSG)
Abbreviations: BMI, body mass index; SBP, systolic blood pressure; DBP, diastolic blood pressure; HSI, hepatic steatosis index; NAFLD, non-alcoholic fatty liver disease; ESG, endoscopic sleeve gastroplasty; LSG, laparoscopic sleeve gastrectomy

**Table S5**: Gastrointestinal symptoms, quality of life, body composition and pathology measures of ESG and LSG participants at baseline, 6-month and 12-month follow-up.

| **Outcome variables** | **ESG** | | | | | | LSG | | | | | |
| --- | --- | --- | --- | --- | --- | --- | --- | --- | --- | --- | --- | --- |
|  | **Baseline** | | **6-months** | | **12-months** | | **Baseline** | | **6-months** | | **12-months** | |
|  | **n** | **Mean (SD)** | **n** | **Mean (SD)** | **n** | **Mean (SD)** | **n** | **Mean (SD)** | **n** | **Mean (SD)** | **n** | **Mean (SD)** |
| **Gastrointestinal symptoms^1^** | | | | | | | | | | | | |
| **Total GSRS** | 15 | 79.2 (17.6) | 9 | 87.4 (6.3) | 4 | 82.0 (16.3) | 43 | 79.5 (13.9) | 34 | 80.9 (18.4) | 27 | 87.0 (11.9) |
| Indigestion | 15 | 6.6 (2.3) | 9 | 9.0 (0.9) | 4 | 7.2 (2.1) | 43 | 7.1 (2.2) | 34 | 7.6 (2.3) | 27 | 8.3 (1.7) |
| Diarrhea | 15 | 8.4 (2.6) | 9 | 9.0 (1.2) | 4 | 9.0 (1.2) | 43 | 8.3 (1.8) | 34 | 8.5 (2.5) | 27 | 8.9 (1.4) |
| Constipation | 15 | 8.3 (2.7) | 9 | 8.7 (1.2) | 4 | 7.8 (3.9) | 43 | 8.3 (1.7) | 34 | 8.1(2.2) | 27 | 8.7(1.6) |
| Pain | 15 | 8.2 (1.9) | 9 | 8.6 (0.9) | 4 | 8.5 (1.7) | 43 | 8.1 (1.5) | 34 | 8.4 (2.0) | 27 | 8.9 (1.2) |
| Reflux | 15 | 8.9 (2.1) | 9 | 9.2 (1.0) | 4 | 10.0 (0.0) | 43 | 8.3 (2.3) | 34 | 8.3 (2.3) | 27 | 8.9(2.1) |
| **Quality of Life^2^** | | | | | | | | | | | | |
| **Total IWQOL** | 13 | 58.0 (14.6) | 9 | 78.2 (15.9) | 4 | 77.8 (28.2) | 42 | 41.5 (14.2) | 34 | 79.3 (16.8)* | 27 | 89.6 (9.7)* |
| Physical function | 14 | 56.2 (21.0) | 9 | 84.8 (13.8) | 4 | 84.2 (21.3) | 43 | 44.4 (18.8) | 34 | 83.1 (17.4) | 27 | 91.8 (7.9) |
| Self-esteem | 14 | 32.6 (22.7) | 9 | 60.3 (29.9) | 4 | 62.5 (32.6) | 43 | 18.6 (16.7) | 34 | 66.9 (19.7) | 27 | 80.2 (19.2) |
| Sexual life | 14 | 57.1 (29.9) | 9 | 74.3 (26.3) | 4 | 73.5 (49.1) | 42 | 39.8 (22.8) | 32 | 74.6 (23.9) | 27 | 86.1 (17.3) |
| Public distress | 14 | 76.1 (15.6) | 9 | 83.9 (17.3) | 4 | 85 (23.8) | 42 | 52.7 (24.3) | 34 | 85.7 (19.4) | 27 | 96.3 (7.0) |
| Work | 13 | 73.5 (17.1) | 9 | 87.6(16.9) | 4 | 83 (30.1) | 41 | 61.1 (23.1) | 34 | 87.7 (19.5) | 27 | 94.5 (9.7) |
| **Body composition^3^** | | | | | | | | | | | | |
| Fat mass, kg | 10 | 48.5 (8.7) | 8 | 37.1 (2.8)^ | 4 | 35.5 (4.8) | 34 | 57.9 (13.3) | 33 | 36.6 (10.4)* | 25 | 32.2 (10.1)* |
| Fat-free mass, kg | 10 | 49.7 (9.9) | 8 | 50.0 (12.8)^ | 4 | 46.7 (2.7)^ | 34 | 53.1 (9.7) | 33 | 47.5 (8.4)* | 25 | 48.3 (9.2)* |
| Bone mineral content, kg | 10 | 2.8 (0.3) | 8 | 3.0 (0.7) | 4 | 2.8 (0.2) | 34 | 2.8 (0.5) | 33 | 2.8 (0.5)* | 25 | 2.8 (0.5)* |
| Android/Gynoid fat mass ratio | 10 | 1.08 (0.14) | 8 | 1.05 (0.13) | 4 | 1.01 (0.16) | 34 | 1.13 (0.17) | 33 | 1.08 (0.15)* | 25 | 1.04 (0.21)* |
| **Pathology**^4^ | | | | | | | | | | | | |
| Fasting blood glucose (mmol/L) | 16 | 5.1 (0.4) | 7 | 4.6 (0.2) | 5 | 4.6 (0.2) | 43 | 5.4 (1.2) | 33 | 4.8 (0.5)* | 20 | 5.0 (0.5)* |
| HbA1c (%) | 16 | 5.0 (0.3) | 6 | 4.8 (0.2) | 5 | 4.7 (0.3)^ | 24 | 5.3 (0.5) | 28 | 5.1 (0.5)* | 13 | 5.2 (0.7)* |
| Total cholesterol (mmol/L) | 16 | 5.6 (0.8) | 7 | 4.6 (0.7) | 5 | 4.5 (0.6) | 44 | 5.1 (1) | 33 | 5.0 (1.0) | 20 | 4.7 (1.0) |
| LDL cholesterol (mmol/L) | 13 | 3.4 (0.6) | 7 | 2.8 (0.6) | 5 | 2.8 (0.6) | 40 | 3.3 (0.9) | 32 | 3.1 (1.1) | 18 | 2.9 (1.0) |
| HDL cholesterol (mmol/L) | 13 | 1.6 (0.5) | 7 | 1.3 (0.4) | 5 | 1.2 (0.5) | 40 | 1.2 (0.3) | 32 | 1.3 (0.3) | 18 | 1.4 (0.4)* |
| Triglycerides (mmol/L) | 16 | 1.4 (0.9) | 7 | 0.9 (0.3) | 5 | 0.8 (0.3) | 43 | 1.4 (0.7) | 33 | 1.1 (0.5) | 20 | 1.0 (0.5)* |
| ALT (U/L) | 16 | 32.5 (20.1) | 7 | 17.7 (7.8) | 5 | 22.2 (10.3) | 45 | 33.3 (20.6) | 33 | 20.6 (11.6) * | 20 | 23.4 (7.5) * |
| AST (U/L) | 16 | 26.4 (9.4) | 7 | 18.3 (4.5) | 5 | 22.0 (6.0)^ | 44 | 24.9 (10.5) | 33 | 18.4 (4.7)* | 20 | 22.2 (7.8)* |
| Albumin (g/L) | 16 | 38.9 (7.5) | 7 | 39.1 (1.3) | 5 | 40.4 (3.4) | 44 | 39.8 (4.2) | 32 | 40.1 (2.7) | 19 | 41.4 (7.3) |
| HSI score | 16 | 46.8 (6.3) | 7 | 39.6 (4.1)^ | 5 | 38.5 (5.3)^ | 44 | 52.8 (6.7) | 33 | 41.5 (7.5)* | 19 | 39.4 (5.2)* |
| **Pathology indicating comorbidity risk factor** | **n** | **Abnormal ^5^ (%)** | **n** | **Abnormal ^5^ (%)** | **n** | **Abnormal ^5^ (%)** | **n** | **Abnormal ^5^ (%)** | **n** | **Abnormal ^5^ (%)** | **n** | **Abnormal ^5^ (%)** |
| Fasting blood glucose (mmol/L) | 18 | 6 | 7 | 0 | 5 | 0 | 48 | 8 | 33 | 3 | 20 | 5 |
| HbA1c (%) | 16 | 0 | 6 | 0 | 5 | 0 | 27 | 0 | 28 | 0 | 13 | 0 |
| Total cholesterol (mmol/L) | 18 | 44 | 7 | 0 | 5 | 0 | 49 | 31 | 33 | 30 | 20 | 20 |
| LDL cholesterol (mmol/L) | 14 | 21 | 7 | 0 | 5 | 0 | 42 | 12 | 32 | 19 | 18 | 17 |
| HDL cholesterol (mmol/L) | 14 | 14 | 7 | 14 | 5 | 40 | 42 | 12 | 32 | 3 | 18 | 17 |
| Triglycerides (mmol/L) | 18 | 22 | 7 | 0 | 5 | 0 | 47 | 21 | 33 | 3^ | 20 | 5 |
| ALT (U/L) | 18 | 11 | 7 | 0 | 5 | 0 | 50 | 24 | 33 | 3 | 20 | 0 |
| AST (U/L) | 18 | 6 | 7 | 0 | 5 | 0 | 49 | 10 | 33 | 0 | 20 | 5 |
| Albumin (g/L) | 18 | 6 | 7 | 0 | 5 | 0 | 49 | 8 | 32 | 0 | 19 | 0 |

^P<0.05 for the difference between baseline measures and 6-month or 12-month measures for participants undergoing the ESG procedure.

*P<0.05 for the difference between baseline measures and 6-month or 12-month measures for participants undergoing the LSG procedure.

^1^ Gastrointestinal symptoms: 1 participant missing information (ESG), 2 participants missing information (LSG); Indigestion: 1 participant missing information (ESG), 2 participants missing information (LSG); Diarrhea: 1 participant missing information (ESG), 2 participants missing information (LSG); Constipation: 1 participant missing information (ESG), 2 participants missing information (LSG); pain: 1 participant missing information (ESG), 2 participants missing information (LSG); reflux: 1 participant missing information (ESG), 2 participants missing information (LSG).

^2^ Total Quality of life: 3 participants missing information (ESG), 3 participants missing information (LSG); Physical function: 2 participants missing information (ESG), 2 participants missing information (LSG); Self-esteem: 2 participants missing information (ESG), 2 participants missing information (LSG); Sexual life: 2 participants missing information (ESG), 3 participants missing information (LSG); Public distress: 2 participants missing information (ESG), 3 participants missing information (LSG); Work: 3 participants missing information (ESG), 4 participants missing information (LSG).

^3^ Body composition: Baseline: 6 participants missing information (ESG), 11 participants missing information (LSG); 6-month FU: 8 participants missing information (ESG), 12 participants missing information (LSG); 12-month FU: 12 participants missing information (ESG), 20 participants missing information (LSG)

^4^Pathology. Fasting blood glucose: 2 participants missing information (LSG); HbA1c: 1 participant missing information (ESG), 21 missing information (LSG); Total cholesterol: 1 participant missing information (LSG); LDL cholesterol: 3 participants missing information (ESG), 5 participants missing information (LSG); HDL cholesterol: 3 participants missing information (ESG), 5 participants missing information (LSG); Triglycerides: 2 participants missing information (LSG); AST: 1 participant missing information (LSG); Albumin:1 participant missing information (LSG); HSI score: 1 participant missing information (LSG).

^5^ Percent of participants with comorbidity indicator-associated biochemistry values outside the normal range (Y/N), with abnormal values defined as: Fasting blood glucose >6.0 mmol/L X, HbA1c >6.4 %, Total cholesterol >5.5 mmol/L, LDL cholesterol >4.0 mmol/L, HDL cholesterol <0.9 mmol/L, Triglycerides >2.0 mmol/L, ALT >46 U/L, AST >41 U/L, Albumin <34 g/L.


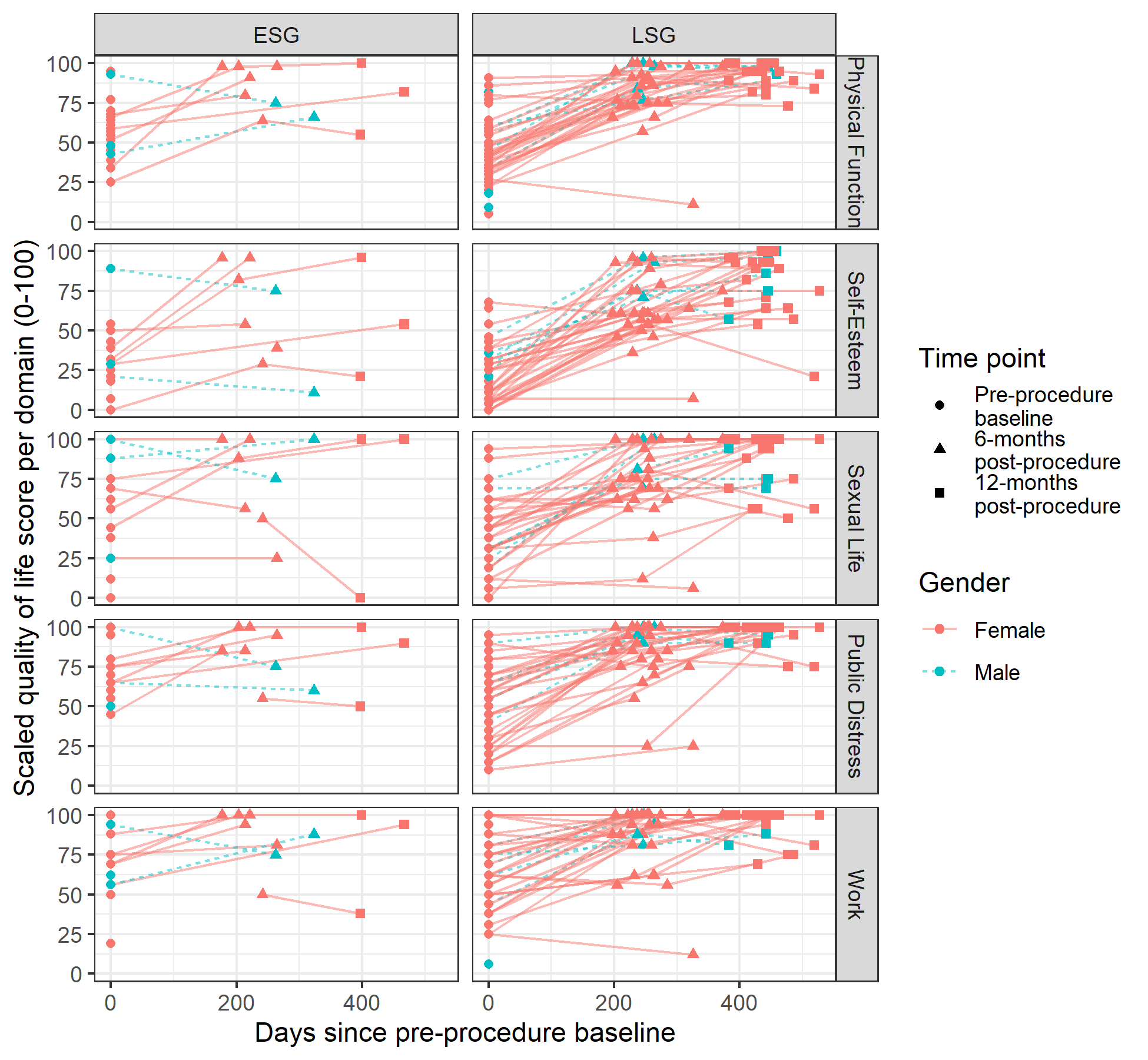
**Figure S1.** Weight related quality of life score by domain of study participants at baseline, 6-months and 12-months follow-up undergoing either ESG or LSG procedure.

**Table S6**: Non-gastrointestinal symptom-related adverse event etiology, severity, expectedness, relatedness, and treatment which occurred in adults who elected ESG and LSG procedures from day of surgery to 12-months post-procedure.

| **Event** | **Timepoint** | **Etiology** | **Severity** | **Expectedness** | **Relatedness** | **Treatment** |
| --- | --- | --- | --- | --- | --- | --- |
| **ESG Cohort** | | | | | | |
| Suspected allergic reaction to procedure-related medication with symptoms of mild erythema, distal swelling to elbow and heat. | 2-weeks  post-procedure | Procedure-related medication (suspected), unknown pre-existing allergy | Moderate | Unexpected | Possibly | Ceased procedure-related medication and overnight observation. |
| Vasovagal syncope and fall causing fracture and laceration to nose | 2-weeks  post-procedure | Dehydration (suspected) | Moderate | Expected | Possibly | Presentation to hospital for intravenous rehydration, stitches to notes, CT of face. |
| Swelling to the face and arm. | 2-weeks  post-procedure | Unknown | Moderate | Unexpected | Possibly | Referral to proceduralist for follow-up. |
| Hair loss | 6-months post-procedure | Protein deficiency | Mild | Expected | Possibly | Referral to dietitian; nutrient supplementation, dietary advice |
| **LSG Cohort** | | | | | | |
| Pancreatitis and jaundice | 2-weeks post-procedure | Biliary sludge plus historical gall bladder removal | Serious | Unexpected | Possibly | Presentation to hospital; conservative management |
| Pain in upper right quadrant | 2-weeks  post-procedure | Unspecified | Mild | Expected | Possibly | Analgesic |
| Dehydration | 2-weeks  post-procedure | Inadequate fluid intake | Serious | Expected | Possibly | Advised to present to hospital for intravenous fluids |
| Intermittent pain on left side of abdomen and chest | 2-weeks  post-procedure | Not specified | Mild | Expected | Possibly | Not specified |
| Stitch pain | 2-weeks  post-procedure | Not specified | Mild | Expected | Possibly | Reassurance |
| Taste disturbance; metallic taste | 2-weeks  post-procedure | Not specified | Mild | Expected | Possibly | Not specified |
| Hair loss | 2-weeks  post-procedure | Not specified | Mild | Expected | Possibly | Not specified |
| Disturbed vision with dizziness | 6-months post-procedure | Unknown | Mild | Unexpected | Possibly | Referred to general practitioner for investigations |
| Fatigue | 6-months post-procedure | Unknown | Mild | Expected | Possibly | Referral to dietitian, nutrient supplementation, dietary advice |
| Occasional headache | 6-months post-procedure | Suspected dehydration | Mild | Expected | Possibly | Advice to improve fluid intake; advised to see general practitioner if persists. |
| Pain (unspecified) | 6-months post-procedure | Unknown | Mild | Expected | Possibly | Not specified |
| Gallstones | 12-months post-procedure | Unknown | Moderate | Expected | Possibly | Presentation to hospital; surgical removal of gallbladder |

1. [↑](#endnote-ref-1)
